# Selective ^13^C labelling reveals the electronic structure of flavocoenzyme radicals

**DOI:** 10.1038/s41598-021-97588-7

**Published:** 2021-09-14

**Authors:** Erik Schleicher, Stephan Rein, Boris Illarionov, Ariane Lehmann, Tarek Al Said, Sylwia Kacprzak, Robert Bittl, Adelbert Bacher, Markus Fischer, Stefan Weber

**Affiliations:** 1 Institut für Physikalische Chemie, Albert-Ludwigs-Universität Freiburg, Albertstr. 21, 79104 Freiburg, Germany; 2 Institut für Lebensmittelchemie, Universität Hamburg, Grindelallee 117, 20146 Hamburg, Germany; 3 Fachbereich Physik, Freie Universität Berlin, Arnimallee 14, 14195 Berlin, Germany; 4 Department Chemie, Technische Universität München, Lichtenbergstr. 4, 85748 Garching, Germany; 5 Present Address: Bruker BioSpin GmbH, Silberstreifen 4, 76287 Rheinstetten, Germany

**Keywords:** Biophysical chemistry, Photocatalysis, Chemical physics

## Abstract

Flavocoenzymes are nearly ubiquitous cofactors that are involved in the catalysis and regulation of a wide range of biological processes including some light-induced ones, such as the photolyase-mediated DNA repair, magnetoreception of migratory birds, and the blue-light driven phototropism in plants. One of the factors that enable versatile flavin-coenzyme biochemistry and biophysics is the fine-tuning of the cofactor’s frontier orbital by interactions with the protein environment. Probing the singly-occupied molecular orbital (SOMO) of the intermediate radical state of flavins is therefore a prerequisite for a thorough understanding of the diverse functions of the flavoprotein family. This may be ultimately achieved by unravelling the hyperfine structure of a flavin by electron paramagnetic resonance. In this contribution we present a rigorous approach to obtaining a hyperfine map of the flavin’s chromophoric 7,8-dimethyl isoalloxazine unit at an as yet unprecedented level of resolution and accuracy. We combine powerful high-microwave-frequency/high-magnetic-field electron–nuclear double resonance (ENDOR) with ^13^C isotopologue editing as well as spectral simulations and density functional theory calculations to measure and analyse ^13^C hyperfine couplings of the flavin cofactor in DNA photolyase. Our data will provide the basis for electronic structure considerations for a number of flavin radical intermediates occurring in blue-light photoreceptor proteins.

## Introduction

**Figure 1:**
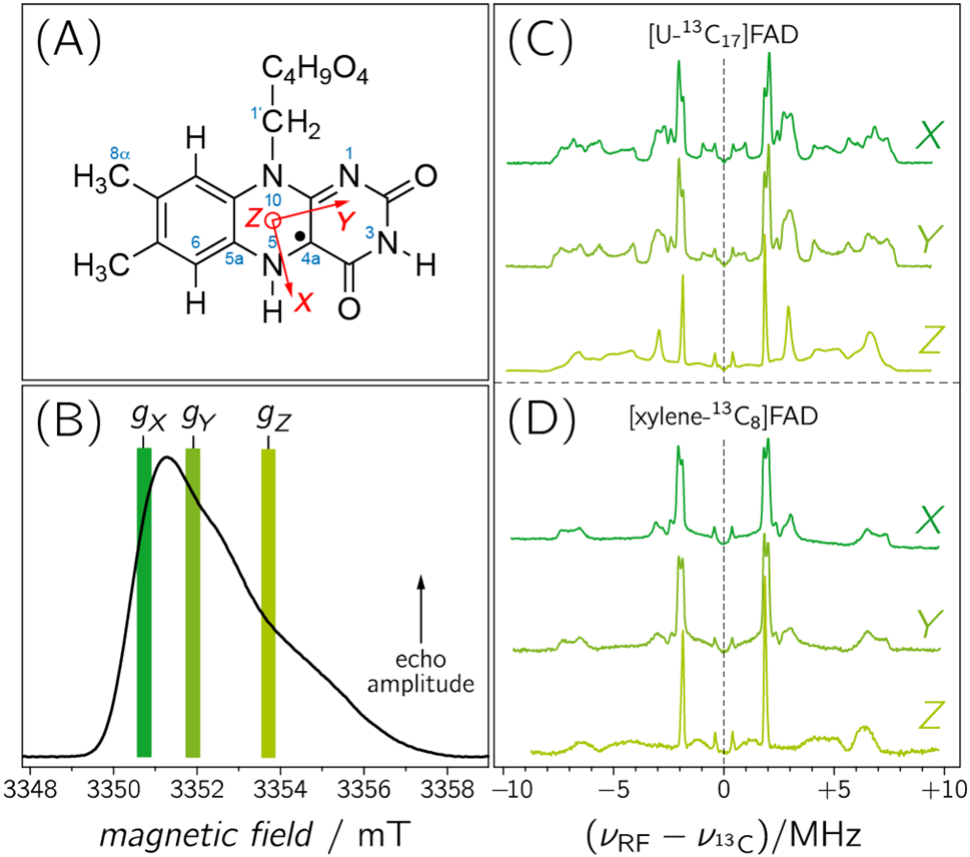
(**A**) IUPAC numbering of riboflavin (including the principal axes of the **g**-matrix). (**B**) Pulsed EPR spectrum of photolyase recorded with a microwave frequency of 94.0 GHz at a temperature of 80 K. The magnetic-field positions of the three principal values of the **g**-matrix (*g*_*X*_ = 2.00429(2), *g*_*Y*_ = 2.00359(2), and *g*_*Z*_ = 2.00218(2)), at which ENDOR spectra were recorded (dark green at *g*_*X*_ , medium green at *g*_*Y*_ and light green at *g*_*Z*_, respectively), were taken from the literature. (**C**) and (**D**): Frozen-solution Davies-type ENDOR spectra of photolyase reconstituted with [U-^13^C_17_]FAD (**C**) and [xylene-^13^C_8_]FAD (**D**).

Amino acids surrounding an enzymatic cofactor alter its chemical reactivity by providing an environment required to bind and act upon a specific substrate and by adjusting the electronic properties of the cofactor through weak interactions including hydrogen bonding, dipolar couplings, and π-stacking. To quantify the parameters that modulate the catalytic power of an enzyme is one of the most fundamental challenges in modern biochemistry and biophysics. In this context, proteins with flavin cofactors are of particular interest because their functions are widespread: Since their discovery in the 1930s^1^ flavins (see Fig. 1A) have been recognized as nearly ubiquitous cofactors that are involved in the catalysis of a wide range of biological redox processes^2,3^. They serve as electron transmitters in electron-transfer processes such as oxidative phosphorylation^4^. Furthermore, flavins can act as mediators between typical two-electron donors such as NADH and one-electron acceptors such as the heme group. The ability of flavins to engage in one- as well as in two-electron-transfer reactions is due to their inherent property of adopting three different redox states^5,6^. Besides their involvement in redox reactions, flavins also catalyse reactions without a net redox change such as blue-light sensing and the repair of UV-light-induced DNA lesions^7–11^. In these processes, the metastable one-electron reduced radical form plays an essential role, either as a reaction intermediate^12^ or, in case of receptor functioning, as a putative signalling state^13,14^.

Starting in the early 1980s, considerable effort was invested in solving the puzzle of how flavin cofactors are modulated to optimize their properties for the catalysis of a specific reaction^15^. Artificially altered flavins, such as 8-azidoflavin or 5-deazaflavin, were incorporated into various proteins to replace the native cofactor. This strategy has been proven quite valuable, since cofactor analogues with, e.g. chemically reactive substituents can be used as probes of the flavin environment. However, artificial flavins exhibit different redox potentials, chemical properties and reactivity, and consequently, modify catalytic rates^16^. Therefore, for the study of the specific electronic structure of natural flavin cofactors in a variety of protein environments, a more sensitive and less intrusive method is required, e.g. based on isotope labelling of the cofactor and/or the protein^17,18^.

The most sensitive and accessible probes of the electronic structure of a cofactor in its paramagnetic state are the electron Zeeman interaction and electron–nuclear hyperfine couplings (hfcs). The isotropic contributions of the latter reflect the unpaired electron spin density at the positions of magnetic nuclei (|*A*_iso_| ~|*Ψ*(0)|^2^)^19^. In most cases, these parameters can be precisely measured using electron paramagnetic resonance (EPR) and/or electron–nuclear double resonance (ENDOR). Up to now, however, mostly proton hfcs from different flavoproteins have been exploited for this purpose^20–24^. Unfortunately, hfcs of protons are insufficient to draw a precise picture of the electron-spin density distribution in flavins as most protons are relatively remote from locations of high unpaired electron-spin density in the π-system of the 7,8-dimethyl isoalloxazine moiety, and furthermore, because protons in flavins are relatively scarce. Mapping the unpaired electron-spin density by directly measuring ^13^C hfcs using ENDOR is much more promising, but has been hampered by (i) the low natural abundance of ^13^C, (ii) the limited availability of ^13^C flavin isotopologs, and (iii) the strong overlap of ^13^C hyperfine resonances with those arising from other magnetic nuclei, predominantly nitrogens, in ENDOR spectra recorded with EPR frequencies around 10 GHz, so that an unambiguous signal assignment could not be achieved.

In this contribution we exploit the potential of high-field ENDOR^25–28^ in combination with selective isotopologue editing to overcome the problem of measuring and assigning ^13^C hfcs of a flavin cofactor in its native protein environment. This facilitates a thorough mapping of the hyperfine structure and hence will ultimately allow theoreticians to draw quantitative conclusions on unpaired electron spin densities of flavocoenyzmes in their functional surroundings. The ENDOR signals of ^13^C nuclei are disentangled from resonances of other magnetic nuclei, such as ^1^H and ^14/15^N, at very high magnetic fields where the Larmor frequencies become increasingly different. Under such conditions, the ^13^C hfcs can be analysed uncompromised by spectral overlap. Furthermore, pulsed ENDOR performed at magnetic fields where the **g**-matrix anisotropy of a radical becomes dominant provides improved spectral resolution enabling the selection of molecules with specific orientations with respect to the direction of the external magnetic field even in a non-oriented sample^29,30^. Due to the small **g**-matrix anisotropy typically observed in flavins^31,32^, experiments performed at high magnetic field, high microwave and radio frequencies are expected to provide increased orientation selection effects that allow to evaluate the extent of hyperfine anisotropy.

To obtain unequivocal assignments of the ^13^C ENDOR signals to individual carbons in the 7,8-dimethyl isoalloxazine moiety of the flavin, we recorded pulsed ENDOR spectra at W-band EPR frequencies of a flavoprotein, which had been produced with a variety of flavin isotopologues carrying ^13^C atoms at strategic positions. The flavin adenine dinucleotide (FAD) binding DNA photolyase from *Escherichia coli* was chosen as a paradigm system for a proof of concept for several reasons: (i) The established purification procedure directly affords the neutral semiquinone radical, FADH^•^; (ii) the paramagnetic flavin form is a reactive intermediate in the enzymatic DNA-repair cycle of the protein^33^; (iii) high-resolution structure data are available from X-ray crystallography^34,35^; and (iv) flavin radicals in the photolyase/cryptochrome protein family have been proposed as key factors in some animal magnetoreception mechanisms that are believed to be based on correlated radical-pair spin chemistry^13,36,37^.

## Results and discussion

The frozen-solution echo-detected EPR signal of FADH^•^ in *E. coli* DNA photolyase recorded with a microwave frequency of 94.0 GHz is shown in Fig. 1B. The radical signature is clearly asymmetric because the electron-Zeeman anisotropy, determined by the **g**-matrix of FADH^•^, exceeds the inhomogeneous spectral linewidth. By recording ENDOR spectra at different magnetic field positions in the resonance range one can now detect molecules with particular orientations with respect to the direction of the external magnetic field vector ***B***_0_^29,38,39^. For example, recording ENDOR at a magnetic-field strength *B*_0_ corresponding to the principal value *g*_*Z*_ of FADH^•^ reveals hyperfine data of those molecules whose principal ***Z*** axis is aligned parallel to ***B***_0_ thus resembling data from a partially oriented sample. The situation is slightly more intricate at *g*_*Y*_*,* or in case of a **g**-matrix anisotropy approaching axial symmetry (i.e. *g*_*X*_ ≈ *g*_*Y*_) also at *g*_*X*_: the resulting ENDOR spectra become less “pure” and comprise resonances from molecules within a range of orientations predominantly with respect to the **g** principal axes ***X*** and ***Y***. The extent of hyperfine anisotropy and the directions of the principal axes of the hyperfine matrix with respect to those of the **g**-matrix can still be analysed using spectral simulations.

Accordingly, Fig. 1C shows Davies-type pulsed ENDOR data of photolyase produced with uniformly ^13^C-labeled FAD ([U-^13^C_17_]FAD) and recorded at different magnetic field strengths *B*_0_. Within the weak-hfc limit (i.e. |hfc|$\ll$*ν*_13C_), numerous pairs of hyperfine resonances are observed that are symmetrically arranged around the free ^13^C-Larmor frequency *ν*_13C_. The spectra recorded at the three canonical orientations of the **g**-matrix, i.e. at *g*_*X*_, *g*_*Y*_ and *g*_*Z*_, are clearly distinct due to the above-mentioned orientation-selection effects. In sets of such spectra, hfc components of a specific ^13^C nucleus can now be directly extracted from the splittings between associated hyperfine resonances with the aid of spectral simulations (see Supplementary Information).

Despite the additional information from the anisotropy of the hfcs, the resonances arising from 17 ^13^C-hfcs in [U-^13^C_17_]FAD are not readily assigned. To disentangle the rather complex ENDOR spectra of the [U-^13^C_17_]FAD-labelled photolyase, application of selective isotope editing is an obvious requirement. Figure 1D shows ENDOR spectra of the protein reconstituted with [5a,6,7,7α,8,8α,9,9a-^13^C_8_]FAD ([xylene-^13^C_8_]FAD), recorded under the same experimental conditions. As expected, fewer signals are now observed, and by employing quantum-chemical computations of ^13^C hfcs, one could envisage an assignment provided that the theoretical predictions are sufficiently precise. However, the reliability of hyperfine predictions strongly depends on the structural model used, and only in very rare cases do calculated and experimental hfcs of more complex aromatic cofactors, such as flavins, agree to within ± 15% or less^40,41^. Consequently, to achieve an unambiguous assignment of hyperfine resonances, examinations of isotopologues with even fewer ^13^C atoms are desirable. Therefore, a library of FAD isotopologues with two or three strategically positioned ^13^C atoms has been devised and examined by high-magnetic-field/high-microwave-frequency ENDOR.

**Figure 2:**
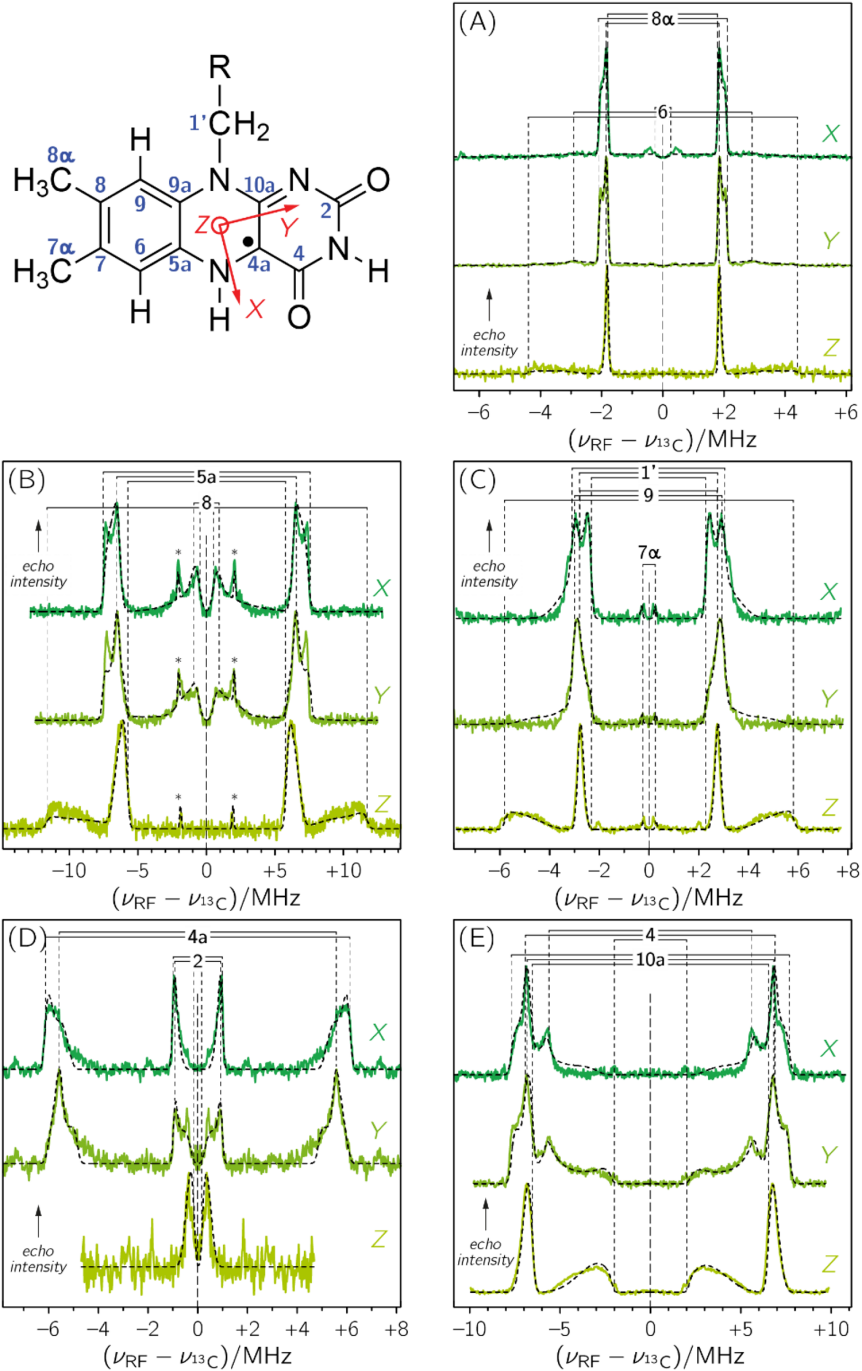
Frozen-solution Davies-type ENDOR spectra of specifically ^13^C-labeled FAD cofactors in *E. coli* photolyase recorded with microwave frequencies around 94 GHz at 80 K: (A) [6,8α-^13^C_2_]FAD, (B) [5a,8-^13^C_2_]FAD (the asterisks denote resonances from the [7,9a-^13^C_2_]FAD isotopomer, see also Fig. S2), (C) [1′,7α,9-^13^C_3_]FAD, (D) [2,4a-^13^C_2_]FAD, and (E) [4,10a-^13^C_2_]FAD. Spectral simulations are depicted as black dashed lines. ENDOR spectra were collected at three magnetic field positions: at *g*_*X*_ (dark green), *g*_*Y*_ (medium green), and *g*_*Z*_ (light green), see also Fig. 1.

As expected, limitation to fewer ^13^C nuclei per sample considerably simplifies ENDOR spectra; Fig. 2 depicts sets of spectra of photolyase reconstituted with various [^13^C]FAD isotopologues: [6,8α-^13^C_2_]FAD (panel A), [5a,8-^13^C_2_]FAD (panel B), [1′,7α,9-^13^C_3_]FAD (panel C), [2,4a-^13^C_2_]FAD (panel D), and [4,10a-^13^C_2_]FAD (panel E). Hyperfine resonances are now sufficiently sparse and well separated such that with the help of (i) a global fit strategy with sophisticated parameter sampling for spectral simulations, (ii) quantum-chemical calculations, (iii) orientation selection of the hyperfine matrix, and (iv) calculations of difference spectra to reduce the complexity of the ENDOR data, a nearly complete hyperfine mapping of all carbons in the isoalloxazine moiety of the flavin can be achieved.

Specifically, ENDOR data at three different magnetic field positions corresponding to *g*_*X*_, *g*_*Y*_ and *g*_*Z*_ were collected for each isotopologue (see Fig. 2). As the principal axes of a hyperfine matrix are determined by the specific bonding situation of the atom, the projection values of the hyperfine matrix (*A*_*X*_, *A*_*Y*_, *A*_*Z*_) can now be extracted with respect to the principal axes (***X***, ***Y***, ***Z***) of the **g**-matrix on the basis of spectral simulations of the ENDOR data. In some equivocal cases, and for obtaining the signs of hfcs, our analysis was aided by quantum-chemical computations. We used density functional theory (DFT) at the B3LYP/EPR-II level of theory to calculate ^13^C-hfcs of the isoalloxazine chromophore bound to photolyase based on the three-dimensional coordinates of the enzyme from X-ray crystallography^34^. Detailed descriptions of signal assignments can be found in the Supplementary Information (Figs. S1 to S5 and Tables S1 to S5). The sets of hyperfine projections arising from C7 and C9a were obtained using difference-ENDOR spectroscopy. For this purpose, the respective orientation-selective ENDOR spectra of photolyases reconstructed with [5a,8-^13^C_2_]FAD, [6,8α-^13^C_2_]FAD and [1′,7α,9-^13^C_3_]FAD were subtracted from those with [xylene-^13^C_8_]FAD as cofactor to obtain ENDOR data of the fictitious [1′,7,9a-^13^C_3_]FAD-containing photolyase (see Fig. S6 and Table S6).

Only the *A*_*Z*_ component of C4a could not be determined by the present strategy: DFT calculations predict very strong anisotropy for the C4a hyperfine matrix, and for its *A*_*Z*_ component values exceeding + 50 MHz. Such strong anisotropy is conducive to spreading signal intensity over a wide radio frequency range, and hence, results in low absolute signal amplitude. Therefore, only two out of the three hyperfine components could be directly measured by ENDOR. We have also applied other pulsed hyperfine methods (such as electron-spin-echo envelope modulation and hyperfine-sublevel correlation at X-band) to detect *A*_*Z*_(C4a) of a neutral flavin radical, but did not obtain unambiguous results.

**Figure 3:**
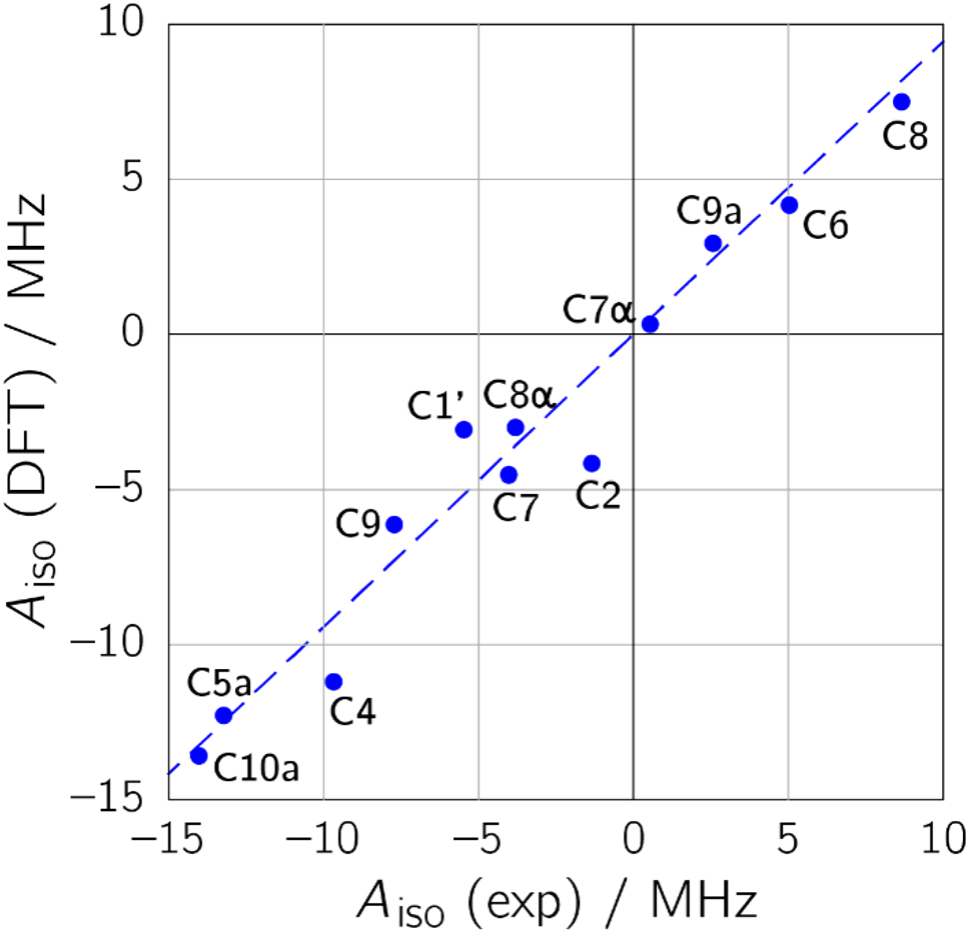
ENDOR/DFT correlation plot for the isotropic ^13^C hyperfine coupling constants of the neutral FAD radical in *E. coli* photolyase. The dashed blue line represents a linear regression fit constrained to go through the origin (*R*^2^ = 0.9576; slope 0.9440).

Finally, the hyperfine projections, *A*_*X*_, *A*_*Y*_, and *A*_*Z*_ were averaged to obtain the isotropic hfc of a particular ^13^C nucleus: *A*_iso_ = (*A*_*X*_ + *A*_*Y*_ + *A*_*Z*_)/3. As the trace of a hyperfine matrix is independent of its coordinate representation, the discussion of this contribution is limited to an interpretation of the *A*_iso_ values. Correlations to DFT-predicted *A*_iso_ values are depicted graphically in Fig. 3 and listed in tabular form in the Supplementary Information (Table S7). Inspection of Fig. 3 reveals that the isotropic hfcs for the various carbons, which are related to the respective electron spin density, span a wide range from −15 to about +10 MHz. Electron spin density is particularly high on the pyrazine ring system of the isoalloxazine moiety, especially at C4, C4a, C5a and C10a (with absolute *A*_iso_ values exceeding 10 MHz). Quite surprisingly, however, rather large hfcs, and hence, high spin densities, are also observed for C1′, C8 and C9. This finding could not be obtained from previous experimental spin density characterizations^22,42,43^ due to the fact that hyperfine data on H6 or H5 only inadequately probe the spin densities at C8 or C9. In contrast to that, rather low electron spin density is detected on C2, C6, C7α, C8α and C9a, which serve as probes in the pyrimidine ring and the xylene ring.

In general, experimental and calculated hfcs are in good agreement, but in some cases, differences clearly exceed 15% of the absolute experimental value even though the immediate protein surroundings of the cofactor within a range of about 7 Å have been taken into account in our computations: For example, calculated hfcs are far too small for position C1′ and far too large for positions C2 and C4. On the other hand, there is no obvious trend of under- or overestimation between calculation and experiment, and the differences for some hfcs, e.g. from C10a and C9a, are very small. We also performed more elaborate structural optimizations that did, however, not lead to a better agreement between experimental and calculated hfcs, see Supplementary Information (Fig. S7 and Table S7). Nevertheless, with accurate experimental hyperfine data being now available it is henceforth possible to calibrate quantum-chemical predictions along the experiment^44,45^.

**Figure 4:**
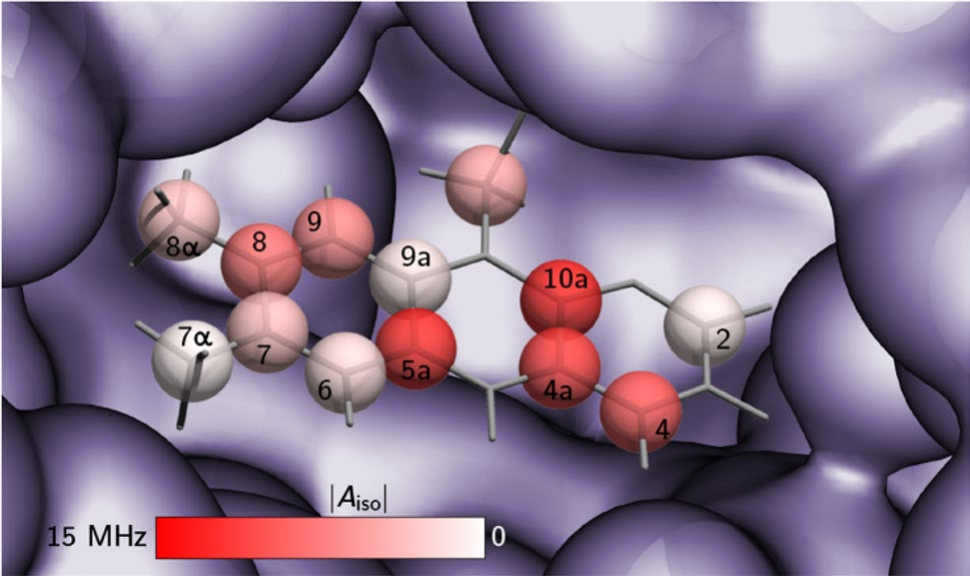
Flavin binding site of *E. coli* photolyase with isotropic ^13^C hfcs obtained from high-magnetic-field ENDOR hyperfine mapping (data were taken from Table S7 in the Supplementary Information). The color intensity reflects the absolute strength of a specific coupling: bright red and white colors depict strong and very weak hfc, respectively. For further details, see experimental section. Protein and cofactor geometries were taken from the PDB: 1DNP.

Figure 4 illustrates the isotropic ^13^C hfcs of the isoalloxazine chromophore of FAD in *E*. *coli* photolyase. The characteristic distribution of the hyperfine pattern can now in principle be used to obtain an optimized electronic wavefunction of the cofactor in its paramagnetic state using quantum-chemical computations, which will then allow correlation to the specific DNA repair activity of the protein. After electron transfer from the light-excited, fully reduced FAD cofactor to the DNA lesion (from the electron-rich pyrazine ring, presumably promoted by the adjacent adenine moiety^46,47^), the resulting flavin-radical intermediate lives for a sufficiently long time so that cyclobutane dimer splitting can take place. Subsequently, the excess electron is pulled back to the FADH^•^ intermediate, thus rendering the reaction “net-zero” with respect to a change of redox states of DNA and FAD. The driving force of this “pulling-back reaction” occurring directly from the DNA back to the FAD radical intermediate^48^ is in the first place mediated by electron densities, but these need to be consistent with the experimental hyperfine structure of the radical. It is quite likely that it is enhanced by the specific electronic structure of DNA photolyases as low electron spin density on the xylene ring is assumed to increase the electron–acceptor probability of the FADH^•^ intermediate. This intermediate in photolyase serves also as a model for the FADH^•^ in spin-correlated radical pairs formed by photoexcitation in the related magnetoreceptor cryptochrome^37,49^, and the hyperfine structure revealed by the present strategy will allow theoreticians to quantitatively evaluate the efficiency of magnetic sensing by taking the specific electronic structure of the radical into account^50,51^.

In summary, the combination of high-field ENDOR with isotopologue editing establishes a basis for a more detailed understanding of the electronic characteristics of flavocoenzymes in general^15^. A thorough hyperfine mapping of various flavoproteins is feasible following our approach and will enable quantitative analyses of reactivity patterns of paramagnetic flavin cofactors. Such a comparison is not restricted to flavin cofactors in their ground state, but will be extended to hyperfine mapping via ENDOR spectroscopy of excited states (e.g. light-generated triplet states). With quantitative information on spin densities at hand, we have now the potential to tackle the fundamental question on the specific role of the protein surrounding on a cofactor and to assess the contributions of electronic versus steric control of a catalysed reaction.

## Methods

### Preparation of selectively labelled FMN

Riboflavin isotopologues were prepared as described elsewhere^52,53^.

### Preparation of DNA photolyase containing selectively labelled FAD

*E. coli* photolyase was overexpressed in *Bacillus subtilis* using a lac-controlled expression system. 5 mg/L of ^13^C-labeled riboflavin was added to vitamin-free Casamino acids medium. Protein was purified by published procedures and stored in liquid nitrogen^54^.

### ENDOR sample preparation

The protein concentration was estimated on the basis of the FAD cofactor’s absorbance at 580 nm (*ε*_580_ = 0.48 × 10^4^ M^–1^ cm^–1^). ENDOR experiments were typically performed with 1.5 mM photolyase in 0.05 M HEPES (pH 7.0) containing 0.1 M KCl and 30% (v/v) glycerol.

### ENDOR instrumentation

Pulsed ENDOR spectra at 94.0 GHz were recorded using a Bruker E680 FT-EPR spectrometer described elsewhere^55^. For Davies-type ENDOR spectroscopy^29^, a π/2 pulse of 120 ns, a separation time *t* of 45 µs and a radio frequency pulse of 40 µs length starting 1 µs after the first microwave pulse were used. The pulse spacing was 500 ns in all experiments. All ENDOR experiments were performed at 80 K.

### ENDOR data analysis

ENDOR raw data were baseline-corrected (polynomial fitting). The spectra were then normalized to a frequency of 94.0 GHz. The orientation-selective ENDOR data were analysed using global least-squares fitting to obtain the best possible agreement between experimental and calculated spectra. The Matlab-based program suite “SpecProFi”^56^ [https://www.radicals.uni-freiburg.de/de/software/specprofi] was used to simultaneously analyse sets of three orientation-selective ENDOR spectra by using one joint set of parameters: typically two or three (depending on the specific [^13^C]FAD isotopologue) sets of hyperfine principal values (*A*_*X*,*i*_, *A*_*Y*,*i*_ and *A*_*Z*,*i*_) and the corresponding sets of Euler angles (*α*_*i*_, *β*_*i*_ and *γ*_*i*_; *z*–*y*–*z*-convention) that relate the principal axes of the respective hyperfine matrix of nucleus *i* to the principal axes system of the flavin’s **g**-matrix^31,57^ (*X*, *Y* and *Z*; see Fig. 1A). Weighting factors have been used to account for the specific isotope enrichment and relaxation properties of the individual ^13^C nucleus under consideration, see Supplementary Information. (The assumption of collinear hyperfine matrices and **g**-matrix principal axes was dropped as it did not yield satisfactory spectral simulations of the experimental data.) Latin hypercube sampling^58,59^, a powerful semi-stochastic algorithm, was invoked to increase the probability of finding the global minimum of the high-dimensional parameter hypersurface by selecting well-distributed initial parameter guesses. Subsequent optimization steps at each of these starting points were conducted by calling the “EasySpin” function “salt”, which calculates powder or single-crystal ENDOR spectra^60^. In all subsequent ENDOR spectral analyses, the **g**-matrix principal values *g*_*X*_ = 2.00431, *g*_*Y*_ = 2.00360, and *g*_*Z*_ = 2.00217, determined from EPR at 360 GHz/12.8 T on the neutral flavin radical in *E. coli* DNA photolyase^31^, were taken as fixed parameters.

### Quantum mechanics calculations of hyperfine couplings

The input structure for the computation of hfcs was based on the crystal structure of *E. coli* photolyase^34^. It was cut down to one layer of amino acids surrounding the FAD cofactor, which itself was truncated to riboflavin. Protons were added, and the resulting structure optimized in a semi-empirical PM3 calculation. DFT computations of hfcs were then carried out using Gaussian03^61^ (Gaussian Inc, Pittsburgh, PA) with the unconstrained compound functional B3LYP, the basis set EPR-II, and a tight model for the self-consistent field method. All resulting hyperfine matrices were then projected to the FADH^•^’s **g**-matrix principal coordinate system^31^ using a self-written MATLAB-routine, see Tables S1 to S6 in the Supplementary Information. The respective hyperfine components were averaged to obtain isotropic hfcs. For Fig. 4, a self-written FORTRAN script was used to generate an input file for POV-Ray rendering. An RGB colour value was calculated corresponding to |*A*_iso_|, with the largest value representing RGB 1–0–0 (red) and the smallest RGB 1–1–1 (white).

## Supplementary Information

Supplementary Information.
