# Supplementary material for: Selective 13C labelling reveals the electronic structure of flavocoenzyme radicals
Source: Sci Rep. 2021 Sep 14;11:18234. doi: 10.1038/s41598-021-97588-7 (PMC8440535; doi:10.1038/s41598-021-97588-7)
Supplement: Supplementary file 1 — Supplementary Information. [file 41598_2021_97588_MOESM1_ESM.pdf]

– Supplementary Information –

Selective ^{13}C labelling reveals the electronic structure of flavocoenzyme radicals

Erik Schleicher[1,*], Stephan Rein[1], Boris Illarionov[2], Ariane Lehmann[1], Tarek Al Said[1], Sylwia Kacprzak[1,+], Robert Bittl[3], Adelbert Bacher[4], Markus Fischer[2], and Stefan Weber[1,*]

General remarks

Errors of hyperfine principal values and Euler angles have been estimated based on single-point calculations, in which the respective optimized parameter value was varied systematically and the resulting simulated ENDOR spectrum compared to the experimental one. The error margins of Euler angles were chosen rather large for nuclei with hyperfine tensors of small overall and axial anisotropy, such as ^{13}C5a, ^{13}C10a and ^{13}C1'.

Weighting factors were necessary to account for the specific isotope enrichment and relaxation properties of the individual ^{13}C nucleus under consideration.

Analysis of ENDOR data from [6,8α-$^{13}C_2$]FAD bound to *Escherichia coli* DNA photolyase

The frozen-solution ENDOR spectra of [6,8α-$^{13}C_2$]FAD-reconstituted *Ec*PL, recorded at magnetic-field values corresponding to the canonical **g**-principal axes orientations, exhibit one pair of intense and narrow signals (resonances "1", see Fig. S1) from which the following components of one hyperfine tensor of nearly axial symmetry could be extracted: $|A_{X,1}|$ = 3.6 MHz, $|A_{Y,1}|$ = 4.2 MHz and $|A_{Z,1}|$ = 3.7 MHz. A much less intense and broader pair of resonances ("2") is also observed that is described with $|A_{X,2}|$ = 0.5 MHz, $|A_{Y,2}|$ = 5.9 MHz and $|A_{Z,2}|$ = 8.9 MHz.

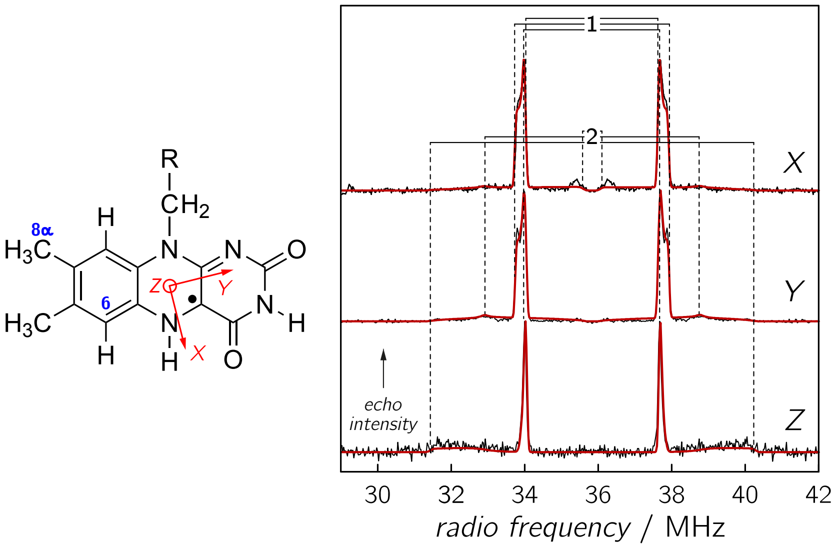

Figure S1. ^{13}C W-band (93.88 GHz) Davies-type ENDOR spectra (black curves) from [6,8α-$^{13}C_2$]FAD-reconstituted *Ec*PL, recorded at 80 K. For improved global least-squares fitting, the data have been symmetrized with respect to the Larmor precession frequency at around 35.85 MHz. For this figure, the spectra have also been normalized to equal maximum intensity with respect to each other. The spectral simulations (red curves) have been obtained with the parameters compiled in Table S1.

From $A_{Y,1}$ being the largest component of hyperfine tensor "1", and from the near axial symmetry $|A_{X,1}| \approx |A_{Z,1}|$, which is typical for the hyperfine coupling tensor of a methyl group that is freely rotating about its C–C bond, even at cryogenic temperatures, we conclude that this set of resonances must arise from ^{13}C8α. This is consistent with predictions from DFT, that yield A_X = –3.0 MHz, A_Y = –3.1 MHz and A_Z = –2.9 MHz. For ^{13}C6, DFT predicts much larger hyperfine anisotropy: A_X = +0.3 MHz, A_Y = +0.8 MHz and A_Z = +11.6 MHz, which is in fair agreement with the simulations of ENDOR resonances "2". All parameters used to obtain the overall simulations shown as red curves in Fig. S1 are compiled in Table S1.

Table S1. Hyperfine data used to simulate the ENDOR spectra of [6,8α-$^{13}C_2$]FAD-reconstituted *Ec*PL. The principal values are accurate within ±0.2 MHz, and the Euler angles within ±0.1 rad. The signs of the hyperfine components were taken from DFT calculations. Γ_G is the full width at half maximum (fwhm) of Gaussian resonance line broadening. The w_N are weighting factors that account, *e. g.*, for the specific relaxation properties of the individual ^{13}C nuclei under consideration.

**Parameter**	**^{13}C6**	**^{13}C8α**
A_X / MHz	(+)0.5	(–)3.6
A_Y / MHz	(+)5.9	(–)4.2
A_Z / MHz	(+)8.9	(–)3.7
a_{iso} / MHz	(+)5.1	(–)3.8
Γ_G / mT	0.07	0.15
α / rad	+1.5	–0.4
β / rad	–0.7	+0.8
γ / rad	−1.8	–0.4
w_N	0.29	0.71

Analysis of ENDOR data from [5a,8-$^{13}C_2$]FAD bound to *Escherichia coli* DNA photolyase

The frozen-solution ENDOR spectra of [5a,8-$^{13}C_2$]FAD-reconstituted *Ec*PL, recorded at magnetic-field values corresponding to the canonical **g**-principal axes orientations, exhibit one pair of intense and relatively narrow signals (resonances "3", see Fig. S2) from which the following components of one hyperfine tensor can be extracted: $|A_{X,3}|$ = 15.1 MHz, $|A_{Y,3}|$ = 13.0 MHz and $|A_{Z,3}|$ = 11.6 MHz. A much weaker and broader pair of resonances ("4") with almost axial symmetry is also observed that is described with $|A_{X,4}|$ = 0.8 MHz, $|A_{Y,4}|$ = 1.8 MHz and $|A_{Z,4}|$ = 23.4 MHz.

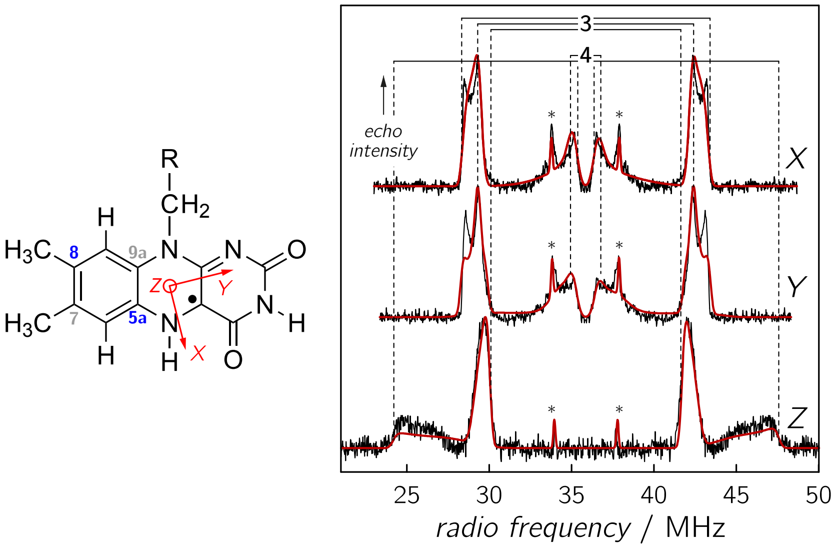

Figure S2. ^{13}C W-band (93.88 GHz) Davies-type ENDOR spectra (black curves) from [5a,8-$^{13}C_2$]FAD-reconstituted *Ec*PL, recorded at 80 K. For improved global least-squares fitting, the data have been symmetrized with respect to the Larmor precession frequency at around 35.85 MHz. For this figure, the spectra have also been normalized to equal maximum intensity with respect to each other. The spectral simulations (red curves) have been obtained with the parameters compiled in Table S2.

For ^{13}C8, DFT predicts a hyperfine tensor of axial symmetry with very large anisotropy, A_X = +1.1 MHz, A_Y = +1.2 MHz and A_Z = +20.1 MHz, which is in good agreement with the ENDOR resonances "4". The predicted hyperfine anisotropy for ^{13}C5a is much smaller: A_X = –10.6 MHz, A_Y = –10.0 MHz and A_Z = –16.2 MHz. Therefore, we assign the resonances "3" to ^{13}C5a.

Each spectrum shown in Fig. S2 contains one pair of resonances (marked with asterisks) that is neither assigned to the hyperfine couplings of ^{13}C8 nor ^{13}C5a. The signals are quite narrow and their position hardly changes on altering the magnetic field value, at which the ENDOR data were recorded. A nearly isotropic hyperfine coupling is extracted if one additional set of

hyperfine principal values is allowed to enter the simulations: $|A_{X,*}| \approx |A_{Y,*}| \approx |A_{Z,*}| \approx |A_{\mathrm{iso},*}|$. This value agrees reasonably well with DFT predictions for ^{13}C7: A_X = –3.8 MHz, A_Y = –3.8 MHz and A_Z = –6.0 MHz. Tentatively we assign the additional hyperfine resonances "*" to ^{13}C7. This notion is corroborated by the determination of the hyperfine coupling of this nucleus using difference ENDOR spectroscopy; see also below. The resonance of $^{13}C_7$ is observed here because in the synthesis procedure of [5a,8-$^{13}C_2$]FAD the [7,9a-$^{13}C_2$]FAD isotopomer is also be formed to some extent.[1] The signal of ^{13}C9a escapes clear detection because its hyperfine tensor is quite anisotropic (see Table S6) and the intensities of its resonances in ENDOR consequently rather low, and therefore, do not exceed the noise level.

All parameters used to obtain the simulations shown as red curves in Fig. S2 are compiled in Table S2.

Table S2. Hyperfine data used to simulate the ENDOR spectra of [5a,8-$^{13}C_2$]FAD-reconstituted *Ec*PL. The principal values are accurate within ±0.2 MHz, and the Euler angles within ±0.1 rad. The signs of the hyperfine components were taken from DFT calculations. Γ_G is the fwhm of Gaussian broadening. The w_N are weighting factors that account, *e. g.*, for the specific relaxation properties of the individual ^{13}C nuclei.

**Parameter**	**^{13}C5a**	**^{13}C8**	**^{13}C7**
A_X / MHz	(–)15.1	(+)0.8	(–)4.1
A_Y / MHz	(–)13.0	(+)1.8	(–)4.1
A_Z / MHz	(–)11.6	(+)23.4	(–)3.7
a_{iso} / MHz	(–)13.2	(+)8.7	(–)4.0
Γ_G / mT	0.35	0.46	0.15
α / rad	+0.2	–0.1	0
β / rad	+0.5	–0.2	0
γ / rad	+0.4	+0.9	0
w_N	0.62	0.35	0.03

Analysis of ENDOR data from [1’,7α,9-$^{13}C_3$]FAD bound to *Escherichia coli* DNA photolyase

The frozen-solution ENDOR spectra of [1’,7α,9-$^{13}C_3$]FAD-reconstituted *Ec*PL, recorded at magnetic-field values corresponding to the canonical **g**-principal axes orientations, exhibit two pairs of quite intense signals (resonances “5” and “6”, see Fig. S3) from which the following components of two hyperfine tensors can be extracted: $|A_{X,5}|$ = 6.1 MHz, $|A_{Y,5}|$ = 5.6 MHz and $|A_{Z,5}|$ = 4.6 MHz, and $|A_{X,6}|$ = 5.5 MHz, $|A_{Y,6}|$ = 5.9 MHz and $|A_{Z,6}|$ = 11.6 MHz. Due to the strong overlap of the resonances, a higher error margin is assumed for these hyperfine components. A much weaker pair of resonances (“7”) with virtually no variation of the splitting with respect to the magnetic-field position is also observed that is described with $|A_{X,7}| \approx |A_{Y,7}| \approx |A_{Z,7}| \approx |A_{\mathrm{iso},7}|$ = 0.5 MHz.

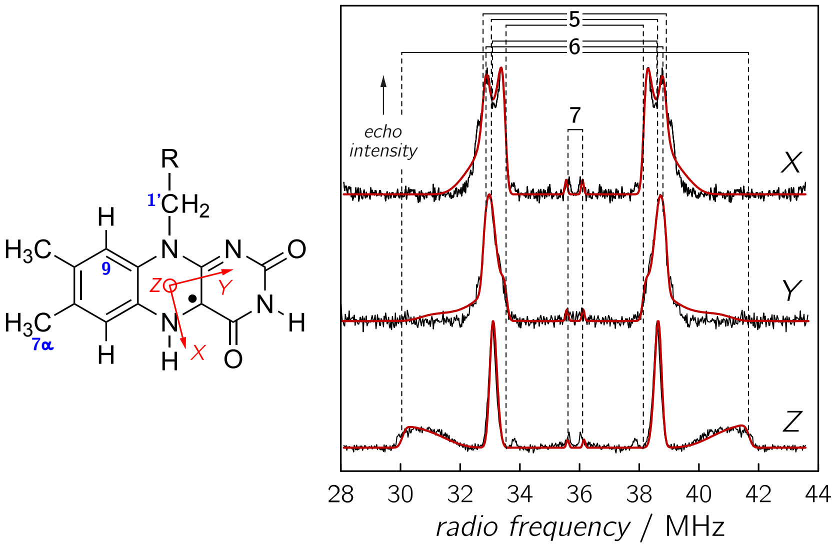

Figure S3. ^{13}C W-band (93.88 GHz) Davies-type ENDOR spectra (black curves) from [1’,7α,9-$^{13}C_3$]FAD-reconstituted *Ec*PL, recorded at 80 K. For improved global least-squares fitting, the data have been symmetrized with respect to the Larmor precession frequency at around 35.85 MHz. For this figure, the spectra have also been normalized to equal maximum intensity with respect to each other. The spectral simulations (red curves) have been obtained with the parameters compiled in Table S3.

For ^{13}C1’, DFT predicts a hyperfine tensor with small anisotropy, A_X = –2.5 MHz, A_Y = –3.5 MHz and A_Z = –3.2 MHz, and for ^{13}C9 one with larger anisotropy and of axial symmetry: A_X = –4.4 MHz, A_Y = –3.9 MHz and A_Z = –10.1 MHz. Therefore, we assign resonances “5” to ^{13}C1’, and resonances “6” to ^{13}C9. Consequently, resonances “7” must arise from ^{13}C7α, for which only small spin-density is predicted from quantum-chemical calculations, as is evident from its nearly isotropic hyperfine tensor: A_X = –0.3 MHz, A_Y = –0.5 MHz and A_Z = –0.1 MHz. All

parameters used to obtain the simulations shown as red curves in Fig. S3 are compiled in Table S3.

Table S3. Hyperfine data used to simulate the ENDOR spectra of [1',7α,9-$^{13}C_3$]FAD-reconstituted *Ec*PL. The principal values of ^{13}C1' are accurate within ±0.2 MHz, the ones from $^{13}C_9$ to ±0.4 MHz, and the ones from ^{13}C7α to ±0.1 MHz. The Euler angles are accurate within ±0.2 rad. The signs of the hyperfine components were taken from DFT calculations. Γ_G is the fwhm of Gaussian broadening. The w_N are weighting factors that account, *e. g.*, for the specific relaxation properties and isotope enrichment of the individual ^{13}C nuclei.

**Parameter**	**^{13}C1'**	**^{13}C9**	**^{13}C7α**
A_X / MHz	(–)6.1	(–)5.5	(–)0.5
A_Y / MHz	(–)5.6	(–)5.9	(–)0.5
A_Z / MHz	(–)4.6	(–)11.6	(–)0.5
a_{iso} / MHz	(–)5.4	(–)7.7	(–)0.5
Γ_G / mT	0.18	0.25	0.10
α / rad	–0.6	+0.8	0
β / rad	–1.2	+0.4	0
γ / rad	–1.2	0.6	0
w_N	0.57	0.42	0.01

Analysis of ENDOR data from [2,4a-$^{13}C_2$]FAD bound to *Escherichia coli* DNA photolyase

The frozen-solution ENDOR spectra of [2,4a-$^{13}C_2$]FAD-reconstituted *Ec*PL, recorded at magnetic-field values corresponding to the canonical **g**-principal axes orientations, reveal two pairs of quite intense signals (resonances "8" and "9", see Fig. S4) from which the following components of two hyperfine tensors can be extracted: $|A_{X,8}|$ = 11.1 MHz and $|A_{Y,8}|$ = 12.2 MHz (a third component could not be determined; the fitting routine revealed a value of 9.6 MHz, which did yield the best possible agreement with the experimental ENDOR data, but this value is certainly an artefact given the limited signal-to-noise ratios of all three spectra), and $|A_{X,9}|$ = 2.0 MHz, $|A_{Y,9}|$ = 1.9 MHz and $|A_{Z,9}|$ = 0.3 MHz.

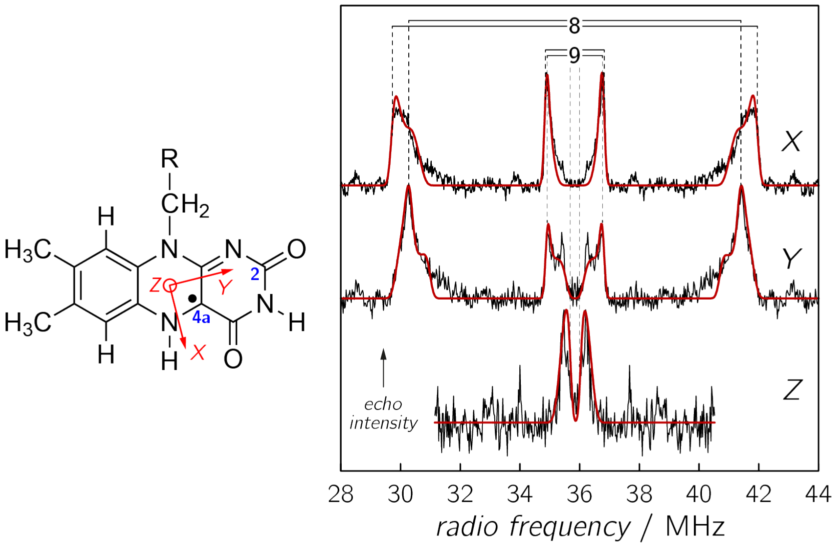

Figure S4. ^{13}C W-band (93.88 GHz) Davies-type ENDOR spectra (black curves) from [2,4a-$^{13}C_2$]FAD-reconstituted *Ec*PL, recorded at 80 K. For improved global least-squares fitting, the data have been symmetrized with respect to the Larmor precession frequency at around 35.85 MHz. For this figure, the spectra have also been normalized to equal maximum intensity with respect to each other. The spectral simulations (red curves) have been obtained with the parameters compiled in Table S4.

DFT predicts a hyperfine tensor with very large anisotropy and axial symmetry for ^{13}C4a, A_X = –10.1 MHz, A_Y = –9.5 MHz and A_Z = +59.2 MHz, and for ^{13}C2 one with much smaller (absolute) hyperfine components: A_X = –4.2 MHz, A_Y = –2.8 MHz and A_Z = –5.7 MHz. Therefore, we assign resonances "9" to ^{13}C2, and resonances "8" to ^{13}C4a. However, the contribution of A_Z to the powder pattern of ^{13}C4a could not be detected in the experimental ENDOR spectrum. DFT predicts values >50 MHz for this principal value. Such strong anisotropy leads to a distribution of signal intensity over a wide radio-frequency range, and hence, very low absolute signal amplitude. Therefore, we exhibit only two out of the three hyperfine components from ENDOR

spectroscopy. All parameters used to obtain the overall simulations shown as red curves in Fig. S4 are compiled in Table S4.

Table S4. Hyperfine data used to simulate the ENDOR spectra of [2,4a-$^{13}C_2$]FAD-reconstituted *Ec*PL. The principal values are accurate within ±0.2 MHz, and the Euler angles within ±0.1 rad. The signs of the hyperfine components were taken from DFT calculations. Γ_G is the fwhm of Gaussian broadening. The w_N are weighting factors that account, *e. g*., for the specific relaxation properties of the individual ^{13}C nuclei.

**Parameter**	**^{13}C2**	**^{13}C4a**
A_X / MHz	(–)2.0	(–)12.2
A_Y / MHz	(–)1.9	(–)11.1
A_Z / MHz	(–)0.3	n.d.
a_{iso} / MHz	(–)1.4	–[a]
Γ_G / mT	0.16	0.22
α / rad	+1.2	−2.1
β / rad	+0.5	+0.6
γ / rad	−0.9	−0.1
w_N	0.309	0.691

[a]Quantum-chemical calculations predict an isotropic hyperfine coupling constant of about +12 MHz for ^{13}C4a. Recent determinations of A_{iso}(^{13}C4a) using photo-CIDNP on the neutral FMN radical in bulk aqueous solution question such a large isotropic hyperfine coupling and suggest values near 0 [N. Pompe *et al*., unpublished data].

Analysis of ENDOR data from [4,10a-$^{13}C_2$]FAD bound to *Escherichia coli* DNA photolyase

The frozen-solution ENDOR spectra of [4,10a-$^{13}C_2$]FAD-reconstituted *Ec*PL, recorded at magnetic-field values corresponding to the canonical **g**-principal axes orientations, reveal two pairs of overlapping powder patterns, one of large anisotropy (resonances "10" , see Fig. S5) and the other of small anisotropy (resonances "11"), from which the following hyperfine components can be extracted: $|A_{X,10}|$ = 11.2 MHz, $|A_{Y,10}|$ = 13.8 MHz and $|A_{Z,10}|$ = 4.0 MHz, and $|A_{X,11}|$ = 15.4 MHz, $|A_{Y,11}|$ = 13.6 MHz and $|A_{Z,11}|$ = 13.1 MHz.

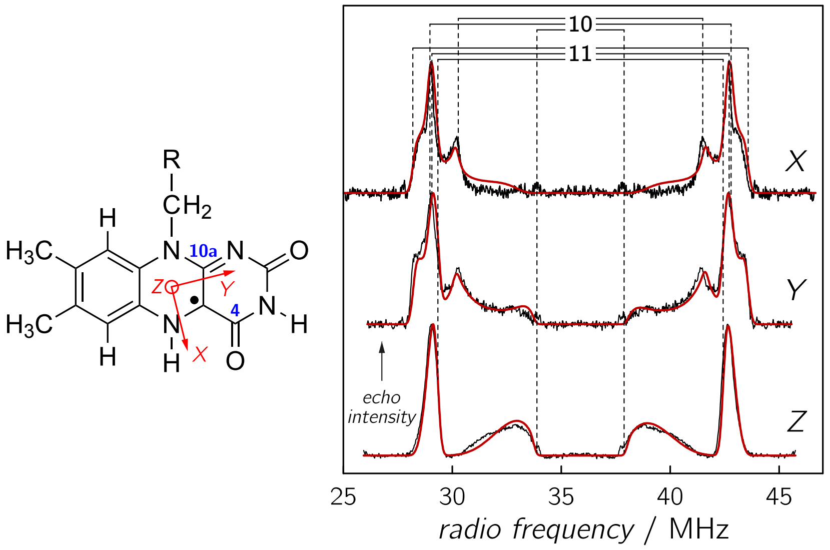

Figure S5. ^{13}C W-band (93.97 GHz) Davies-type ENDOR spectra (black curves) from [4,10a-$^{13}C_2$]FAD-reconstituted *Ec*PL, recorded at 80 K. For improved global least-squares fitting, the data have been symmetrized with respect to the Larmor precession frequency at around 35.88 MHz. For this figure, the spectra have also been normalized to equal maximum intensity with respect to each other. The spectral simulations (red curves) have been obtained with the parameters compiled in Table S5.

For ^{13}C4, DFT predicts a hyperfine tensor with moderate anisotropy, A_X = –11.6 MHz, A_Y = –12.5 MHz and A_Z = –9.4 MHz, and for ^{13}C10a one with smaller anisotropy and of axial symmetry: A_X = –12.5 MHz, A_Y = –14.3 MHz and A_Z = –14.1 MHz. Therefore, we assign resonances "10" to ^{13}C4, and resonances "11" to ^{13}C10a. All parameters used to obtain the overall simulations shown as red curves in Fig. S5 are compiled in Table S5.

Table S5. Hyperfine data used to simulate the ENDOR spectra of [4,10a-$^{13}C_2$]FAD-reconstituted *Ec*PL. The principal values are accurate within ±0.2 MHz, and the Euler angles within ±0.2 rad. The signs of the hyperfine components were taken from DFT calculations. Γ_G is the fwhm of Gaussian broadening. The w_N are weighting factors that account, *e. g*., for the specific relaxation properties of the individual ^{13}C nuclei.

**Parameter**	**^{13}C4**	**^{13}C10a**
A_X / MHz	(–)11.2	(–)15.4
A_Y / MHz	(–)13.8	(–)13.6
A_Z / MHz	(–)4.0	(–)13.1
a_{iso} / MHz	(–)9.7	(–)14.0
Γ_G / mT	0.08	0.37
α / rad	+0.3	+0.3
β / rad	+0.5	+0.6
γ / rad	+1.0	+0.6
w_N	0.49	0.51

Determination of the hyperfine tensors of ^{13}C7 and ^{13}C9a using difference-ENDOR spectroscopy

To determine hyperfine data for the two remaining ^{13}C nuclei in the 7,8-dimethyl isoalloxazine moiety of the neutral flavin radical, ^{13}C7 and ^{13}C9a, we applied difference-ENDOR spectroscopy. Successive subtraction of the ENDOR spectra from *Ec*PL reconstituted with [5a,8-$^{13}C_2$]FAD, [6,8α-$^{13}C_2$]FAD and [1',7α,9-$^{13}C_3$]FAD from the ENDOR spectra of *Ec*PL reconstituted with [xylene-$^{13}C_8$]FAD (= [5a,6,7,7α,8,8α,9,9a-$^{13}C_8$]FAD) results in a set of difference-ENDOR spectra with at the end only positive signals from the remaining nuclei ^{13}C7 and ^{13}C9a, see Fig. S6. The negative signals in Fig. S6 arise from ^{13}C1' absent in [xylene-$^{13}C_8$]FAD and will not be discussed further, since the hyperfine principal values of this nucleus have been determined directly using the [1',7α,9-$^{13}C_3$]FAD isotopolog (see above). Two sets of positive difference-ENDOR amplitudes are observed, resonances "12" and "13". The sets of very narrow lines with a splitting of around 4 MHz are assigned to ^{13}C7. The hyperfine components extracted from the difference ENDOR spectra, $|A_X| = |A_Y| = 4.12$ MHz and $|A_Z| = 3.74$ MHz, agree reasonably well with predictions from DFT for this nucleus: $A_X = -3.8$ MHz, $A_Y = -3.8$ MHz and $A_Z = -6.0$ MHz. (These resonances have also been observed as an "impurity" (resonances marked with "*" in Fig. S2) in the *Ec*PL reconstituted [5a,8-$^{13}C_2$]FAD, see above). The more anisotropic set of hyperfine resonances, "13", is consequently assigned to ^{13}C9a. Due to distortions arising from the overlap with the difference-ENDOR signals from ^{13}C1', its hyperfine components can be read out from the spectra with only moderate accuracy: $|A_X| = 4.8$ MHz, $|A_Y| = 4.6$ MHz, and $|A_Z| = 1.7$ MHz. DFT predicts $A_X = -1.9$ MHz, $A_Y = -1.6$ MHz and $A_Z = +12.2$ MHz for this nucleus.

Table S6. Hyperfine data extracted from the difference-ENDOR spectra of *Ec*PL with "[1',7,9a-$^{13}C_3$]FAD" depicted in Fig. S6. The principal values are accurate within ±0.1 MHz (^{13}C7) and ±1 MHz (^{13}C9a).

**Parameter**	**^{13}C7**	**^{13}C9a**
A_X / MHz	(–)4.1	(–)4.8
A_Y / MHz	(–)4.1	(–)4.6
A_Z / MHz	(–)3.7	(+)1.7
a_{iso} / MHz	(–)4.0	(–)2.6

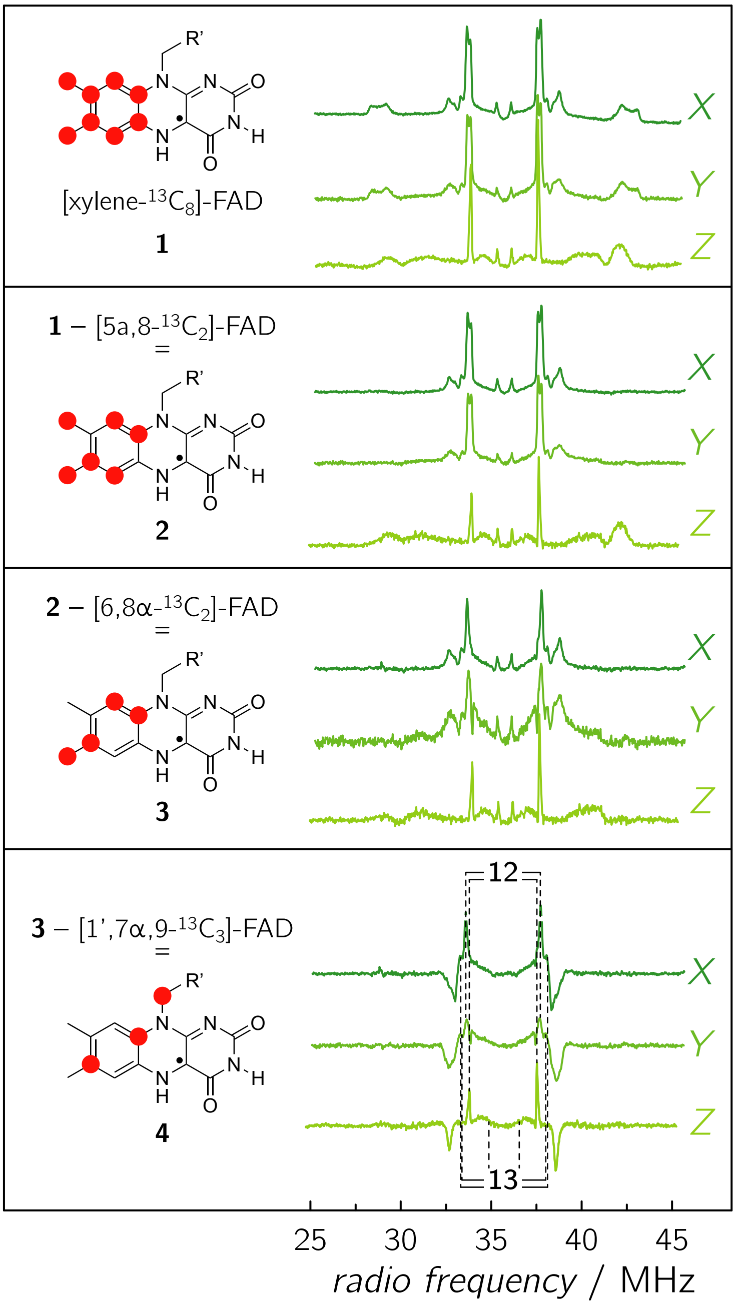

Figure S6. ^{13}C W-band difference-ENDOR data (green curves) from *Ec*PL with the isotopolog species marked on the left sides of the sets of spectra.

Table S7. Compilation of isotropic hyperfine couplings of ^{13}C nuclei in the neutral flavin radical in *Ec*PL from the various ENDOR experiments, DFT calculations based on the PDB structure of *Ec*PL, and DFT calculations following geometry relaxation using QM/MM.

Nucleus	A_{iso} / MHz (experiment)[a]	A_{iso} / MHz (DFT)	A_{iso} / MHz (QM/MM/DFT)[b]
$^{13}C2$	(–)1.4	–4.2	–3.7
$^{13}C4$	(–)9.7	–11.2	–13.1
$^{13}C4a$	–	+13.2	+15.1
$^{13}C5a$	(–)13.2	–12.3	–14.0
$^{13}C6$	(+)5.1	+4.2	+5.0
$^{13}C7$	(–)4.0	–4.5	–4.5
$^{13}C7\alpha$	(+)0.5	+0.3	0.0
$^{13}C8$	(+)8.7	+7.5	+7.7
$^{13}C8\alpha$	(–)3.8	–3.0	–3.3
$^{13}C9$	(–)7.7	–6.1	–5.6
$^{13}C9a$	(+)2.6	+2.9	+0.6
$^{13}C10a$	(–)14.0	–13.6	–17.1
$^{13}C1'$	(–)5.4	–3.1	–2.3

[a]Sign of hyperfine couplings taken from DFT calculations.

[b]To obtain hyperfine data from quantum mechanics/molecular mechanics (QM/MM) computations, we performed in a first step molecular-dynamics (MD) simulations using GROMACS to obtain "equilibrated" structure data for photolyase. The force fields Gromos53a6 and Gromos G96 53a6 united-atoms[2] were used for the protein and the FAD cofactor, respectively. An MD trajectory of 4 ns was computed and representative "snapshots" of the FAD cofactor and its immediate surroundings (comprising the five amino acids Ser-238, Arg-344, Asp-372, Asn-378 and Trp-382) selected that were then used, in the second step, as input coordinates for a QM treatment at the DFT level. Principal values of ^{13}C hyperfine tensors were computed using ORCA with the B3LYP hybrid functional in combination with the EPR-II basis set, and subsequently averaged to yield A_{iso} values.

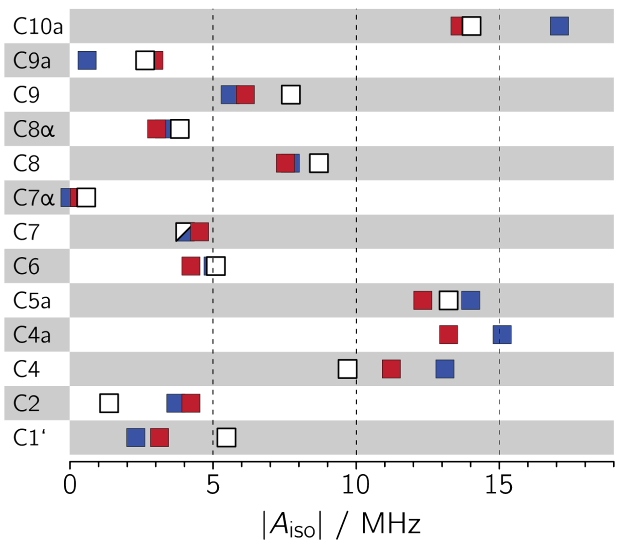

Figure S7. Compilation of (absolute) isotropic ^{13}C hyperfine couplings from ENDOR (white squares), QM/MM/DFT (blue squares) and a "simple" DFT treatment (red squares). Values have been taken from Table S7.

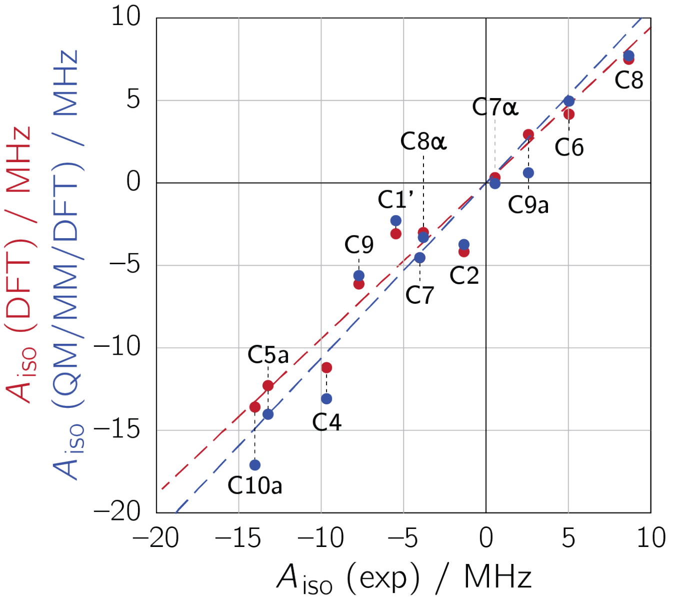

Figure S8. ENDOR/QM/MM/DFT (blue circles) and ENDOR/DFT (red circles) correlation plots for the isotropic ^{13}C hyperfine coupling constants of the neutral FAD radical in *E. coli* photolyase. The dashed blue line (R^2 = 0.9321; slope 1.0619) and the dashed red line (R^2 = 0.9576; slope 0.9440) represent linear regression fits constrained to go through the origin.

References

1 Sedlmaier, H., Müller, F., Keller, P. J. & Bacher, A. Enzymatic synthesis of riboflavin and FMN specifically labeled with ^{13}C in the xylene ring. *Z. Naturforsch. C* **42**, 425-429 (1987).

2 Pauwels, E. *et al.* Influence of protein environment on the electron paramagnetic resonance properties of flavoprotein radicals: a QM/MM study. *J. Phys. Chem. B* **114**, 16655-16665 (2010).
